# High test positivity and low positive predictive value for colorectal cancer of continued faecal occult blood test screening after negative colonoscopy

**DOI:** 10.1177/0969141317698501

**Published:** 2017-05-03

**Authors:** Jeremy P Brown, Kate Wooldrage, Suzanne Wright, Claire Nickerson, Amanda J Cross, Wendy S Atkin

**Affiliations:** 1 Cancer Screening and Prevention Research Group, Department of Surgery and Cancer, Imperial College London, London, UK; 2 NHS Cancer Screening Programmes, Fulwood House, Sheffield, UK

**Keywords:** Colorectal cancer, gFOBT, colonoscopy, screening programme, faecal occult blood test

## Abstract

**Objectives:**

The English Bowel Cancer Screening Programme offers biennial guaiac faecal occult blood test (gFOBT) screening to 60–74-year-olds. Participants with positive results are referred for follow-up, but many do not have significant findings. If they remain age eligible, these individuals are reinvited for gFOBT screening. We evaluated the performance of repeat screening in this group.

**Methods:**

We analysed data on programme participants reinvited to gFOBT screening after either previous negative gFOBT (*n* = 327,542), or positive gFOBT followed by a diagnostic investigation negative for colorectal cancer (CRC) or adenomas requiring surveillance (*n* = 42,280). Outcomes calculated were uptake, test positivity, yield of CRC, and positive predictive value (PPV) of gFOBT for CRC.

**Results:**

For participants with a previous negative gFOBT, uptake in the subsequent screening round was 87.5%, positivity was 1.3%, yield of CRC was 0.112% of those adequately screened, and the PPV of gFOBT for CRC was 9.1%. After a positive gFOBT and a negative diagnostic investigation, uptake in the repeat screening round was 82.6%, positivity was 11.3%, CRC yield was 0.172% of participants adequately screened, and the PPV of gFOBT for CRC was 1.7%.

**Conclusion:**

With high positivity and low PPV for CRC, the suitability of routine repeat gFOBT screening in two years among individuals with a previous positive test and a negative diagnostic examination needs to be carefully considered.

## Introduction

The English Bowel Cancer Screening Programme (BCSP) currently offers screening using the guaiac faecal occult blood test (gFOBT), which has been shown in randomized controlled trials to reduce colorectal cancer cause-specific mortality.^1^ In gFOBT or faecal immunochemical test (FIT) screening programmes, many participants with a positive test do not have cancer or adenomas requiring surveillance. In the BCSP, in line with the British Society of Gastroenterology guidelines, only individuals with intermediate risk adenomas (three or four small, <1 cm diameter adenomas, or at least one adenoma ≥ 1 cm) or high risk adenomas (five or more adenomas, or three or more with at least one ≥ 1 cm) require surveillance following polypectomy.^2^ In the first round of the BCSP, only 37.4% of 17,518 participants undergoing diagnostic investigation (98.1% had colonoscopy as first investigation performed) for positive gFOBT were diagnosed with colorectal cancer or intermediate/high risk adenomas. For the remaining participants undergoing diagnostic investigation, the outcome was normal colon and rectum in 29.7%, low risk adenomas (one or two adenomas <1 cm diameter) in 15.7%, other abnormal colorectal findings in 11.5%, and missing in 5.8%.^3^

In the BCSP, individuals with a positive gFOBT who are referred for diagnostic investigation and found to have a normal colon and rectum, low risk adenomas or other abnormal colorectal findings are currently reinvited to gFOBT screening in two years, provided they remain within the eligible age range (60–74). The performance of repeat gFOBT screening in these three groups is not well understood. Given that these individuals have had a colonoscopy, or other diagnostic investigation, negative for colorectal cancer and intermediate/high risk adenomas, positivity and yield of a repeat gFOBT screen after two years may be low. There is, however, a small miss rate of colonoscopy for both large adenomas and cancer.^4–6^ A repeat gFOBT screen may detect these missed lesions.

A number of studies have previously examined the question of whether further gFOBT or FIT screening is effective in individuals who have had a colonoscopy negative for cancer or adenomas requiring surveillance; however, their conclusions have been contradictory.^7–15^ Furthermore, these previous studies have been based on smaller datasets and have not examined the effect of age, sex, and previous diagnostic test outcome (normal, low risk adenomas, or other abnormal findings) on efficacy of repeat screening.

This investigation aimed to evaluate the outcomes of repeat gFOBT screening in BCSP participants who previously had a positive gFOBT, followed by a diagnostic investigation negative for intermediate or high risk adenomas and colorectal cancer.

## Methods

The BCSP has been described in detail elsewhere.^3^ From the age of 60–74 (previously 60–69), men and women registered to a general practice are invited by post every two years to complete and return a gFOBT kit.

The gFOBT (Hema-Screen®) includes six windows for two samples from three separate stools. No dietary restrictions are requested of participants. If 5–6 windows of the gFOBT are positive, the test is considered positive. When 1–4 windows test positive, participants are immediately invited to take a second test. If any of the windows is positive on retesting, the gFOBT is considered positive. When no windows are positive on retesting, the participant is promptly invited to take a third gFOBT. Similarly, this test is considered positive if any windows test positive. If all windows are negative on either the first or third kit, the subject is considered normal, and discharged from the screening round.

**Figure 1.**
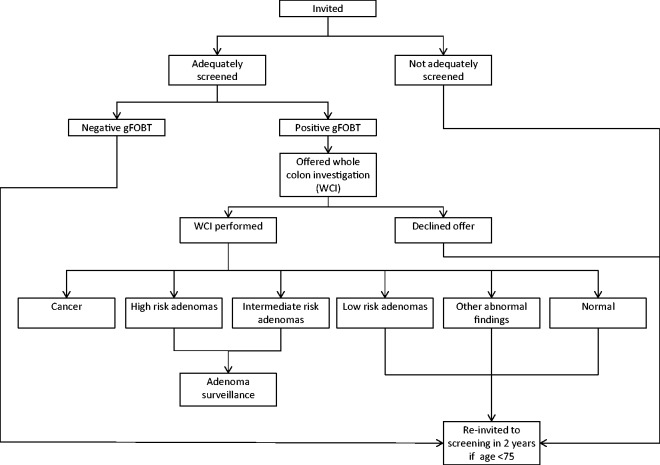
Flow chart of the BCSP screening pathway.

Participants with a positive gFOBT are referred to a specialist screening practitioner (Figure 1). The first line diagnostic investigation for a positive gFOBT is a colonoscopy. In a small proportion of participants (typically < 3%), a colonoscopy is considered inappropriate, and computed tomographic (CT) colonography or barium enema are performed.^3^ The outcome of diagnostic investigation may be normal colon and rectum, low risk adenomas, intermediate risk adenomas, high risk adenomas, cancer, or other abnormal colorectal findings such as diverticular disease, ulcerative colitis, or haemorrhoids.^3^ Following polypectomy, participants who had intermediate or high risk adenomas enter colonoscopic surveillance.

Participants found to have a normal colon and rectum, low risk adenomas or other abnormal colorectal findings at diagnostic investigation are re-invited to gFOBT screening in two years, provided they remain in the eligible age range. We investigated the outcome of repeat gFOBT screening in these people.

We obtained individual-level de-identified data, extracted from the Bowel Cancer Screening System, on 42,280 BCSP participants invited to two consecutive screening rounds between August 2006 and April 2013, where the outcome of the earlier screening round was positive gFOBT and diagnostic investigation negative for cancer and intermediate/high risk adenomas. We compare our findings to aggregate data provided by the BCSP on 327,542 participants invited to a further screening round between January and May 2013 after a negative gFOBT kit in their first screening round.

The key outcome measures included are uptake (participants adequately screened/individuals invited), test positivity (participants with positive gFOBT/participants adequately screened), positive predictive value (PPV) for colorectal cancer (participants with colorectal cancer/participants attending diagnostic investigation following positive gFOBT), and PPV for intermediate or high risk adenomas (participants with intermediate or high risk adenomas/participants attending diagnostic investigation following positive gFOBT). Additionally of interest are yield of colorectal cancer, and yield of intermediate or high risk adenomas, as a percentage of participants adequately screened. Adequately screened participants are defined as those with a definitive positive or negative gFOBT result. Patients were categorized by their most advanced neoplastic finding. For instance, when intermediate or high risk adenomas were detected concurrently with cancer, the outcome was classified as colorectal cancer. Binomial exact 95% confidence intervals (CIs) are presented for each of the key outcomes.

## Results

The majority of the 42,280 BCSP participants with a previous positive gFOBT and diagnostic investigation negative for cancer and intermediate/high risk adenomas had been investigated by colonoscopy (97.2%), with the remainder receiving one or more of CT colonography, barium enema, or flexible sigmoidoscopy. The outcome of these diagnostic investigations was normal colon and rectum, low risk adenomas, and other abnormal colorectal findings in 17,979, 11,578, and 12,723 participants, respectively (Table 1).

**Table 1.** Outcomes of repeat screening stratified by sex and findings of preceding screening round (*n* = 369,822).

	Outcome of preceding round		Outcome of repeat screening round							
	Result of gFOBT	Result of diagnostic investigation	Invited	Adequately screened (%)	Positive gFOBT (%)	Attended diagnostic investigation	CRC (PPV, %)	IR or HR adenomas (PPV, %)	Yield among adequately screened	
Group									CRC (%)	IR or HR adenomas (%)
Overall	Negative	Not applicable	327,542	286,492 (87.5)	3851 (1.3)	3521	321 (9.1)	902 (25.6)	0.112	0.315
	Positive	No cancer or IR/ HR adenomas	42,280	34,942 (82.6)	3940 (11.3)	3572	60 (1.7)	251 (7.0)	0.172	0.718
	Positive	Normal colon & rectum	17,979	14,471 (80.5)	1590 (11.0)	1423	19 (1.3)	77 (5.4)	0.131	0.532
	Positive	Low risk adenomas	11,578	10,014 (86.5)	1100 (11.0)	1029	23 (2.2)	109 (10.6)	0.230	1.088
	Positive	Other abnormal	12,723	10,457 (82.2)	1250 (12.0)	1120	18 (1.6)	65 (5.8)	0.172	0.622
Men	Negative	Not applicable	150,863	131,426 (87.1)	2209 (1.7)	2025	207 (10.2)	614 (30.3)	0.158	0.467
	Positive	No cancer or IR/ HR adenomas	22,730	19,069 (83.9)	2181 (11.4)	1972	34 (1.7)	170 (8.6)	0.178	0.891
	Positive	Normal colon & rectum	8665	7124 (82.2)	783 (11.0)	690	9 (1.3)	49 (7.1)	0.126	0.688
	Positive	Low risk adenomas	7301	6334 (86.8)	710 (11.2)	660	16 (2.4)	75 (11.4)	0.253	1.184
	Positive	Other abnormal	6764	5611 (83.0)	688 (12.3)	622	9 (1.4)	46 (7.4)	0.160	0.820
Women	Negative	Not applicable	176,679	155,066 (87.8)	1642 (1.1)	1496	114 (7.6)	288 (19.3)	0.074	0.186
	Positive	No cancer or IR/ HR adenomas	19,550	15,873 (81.7)	1759 (11.1)	1600	26 (1.6)	81 (5.1)	0.164	0.510
	Positive	Normal colon & rectum	9314	7347 (78.9)	807 (11.0)	733	10 (1.4)	28 (3.8)	0.136	0.381
	Positive	Low risk adenomas	4277	3680 (86.0)	390 (10.6)	369	7 (1.9)	34 (9.2)	0.190	0.924
	Positive	Other abnormal	5959	4846 (81.3)	562 (11.6)	498	9 (1.8)	19 (3.8)	0.186	0.392

CRC: colorectal cancer; IR or HR: intermediate or high risk.

Uptake of a repeat gFOBT screen was 82.6% (95% CI: 82.3–83.0%) in participants with a previous diagnostic investigation negative for cancer or intermediate/high risk adenomas (Table 1). In comparison, uptake was 87.5% (95% CI: 87.4–87.6%) in participants invited to a second round of screening following a negative first round gFOBT.

Positivity of gFOBT for participants with a previous diagnostic investigation negative for cancer or intermediate/high risk adenomas was 11.3% (95% CI: 10.9–11.6%). This is much higher than the 1.3% positivity (95% CI: 1.3–1.4%) observed in BCSP participants in a second screening round following a negative gFOBT.

In the repeat screening round for participants with a previous negative diagnostic investigation, the PPV for colorectal cancer was 1.7% (95% CI: 1.3–2.2%) (Table 1). This was considerably lower than the 9.1% (95% CI: 8.2–10.1%) PPV observed following previous negative gFOBT. Similarly, the PPV for intermediate or high risk adenomas was only 7.0% (95% CI: 6.2–7.9%) in participants with a previous negative diagnostic investigation. In contrast, following a previous negative gFOBT, the PPV for intermediate or high risk adenomas was 25.6% (95% CI: 24.2–27.1%).

Though the PPV for colorectal cancer was low in the repeat screening round following positive gFOBT and negative diagnostic investigation, the yield of colorectal cancer, and the yield of intermediate or high risk adenomas, as a proportion of those adequately screened by gFOBT, was higher than after previous negative gFOBT. Yield of colorectal cancer and yield of intermediate or high risk adenomas were 0.172% (95% CI: 0.131–0.221%) and 0.718% (95% CI: 0.632–0.813%) of participants adequately screened, respectively. In comparison, after previous negative gFOBT, the yield of colorectal cancer and yield of intermediate or high risk adenomas were 0.112% (95% CI: 0.100–0.125%) and 0.315% (95% CI: 0.295–0.336%) of participants adequately screened, respectively.

Among the 42,280 individuals with a positive gFOBT followed by a negative diagnostic examination, the repeat screening round identified 60 individuals with colorectal cancer. Data on lesion size were missing for 16 individuals. For the 44 cancers with information on lesion size, median size was 25 mm (interquartile range: 15–35 mm). Of the 60 individuals with cancer, 57 had received a complete colonoscopy in the previous screening round, one had an incomplete colonoscopy, and two had not received a colonoscopy but had received a CT colonography. Among the 58 individuals who underwent colonoscopy, the quality of bowel preparation had been good for 34 (58.6%), adequate for 21 (36.2%), and poor for two individuals (3.4%); this information was missing for one individual.

In analyses stratified by age, sex, or whether the participant was previously diagnosed as having a normal colon and rectum, low risk adenomas, or other abnormal colorectal findings, repeat gFOBT positivity was high and PPV for colorectal cancer was low, in comparison with participants with a previous negative gFOBT (Tables 1 and 2).

**Table 2.** Outcomes of repeat screening by age group in participants with a previous positive gFOBT followed by a diagnostic investigation negative for cancer and intermediate/high risk adenomas (*n* = 42,280).

		Outcome of repeat screening round					
Result of diagnostic investigation in preceding round	Age group^a^ (years)	Invited	Adequately screened (%)	Positive gFOBT (%)	Attended diagnostic investigation	CRC (PPV, %)	IR or HR adenomas (PPV, %)
Normal colon & rectum	60 to <65	6605	5261 (79.7)	546 (10.4)	480	4 (0.8)	27 (5.6)
	65 to <70	7937	6429 (81.0)	748 (11.6)	672	9 (1.3)	36 (5.4)
	70+	3437	2781 (80.9)	296 (10.6)	271	6 (2.2)	14 (5.2)
Low risk adenomas	60 to <65	3686	3164 (85.8)	332 (10.5)	309	8 (2.6)	30 (9.7)
	65 to <70	5147	4446 (86.4)	510 (11.5)	478	9 (1.9)	49 (10.3)
	70+	2745	2404 (87.6)	258 (10.7)	242	6 (2.5)	30 (12.4)
Other abnormal	60 to <65	4341	3460 (79.7)	420 (12.1)	380	2 (0.5)	24 (6.3)
	65 to <70	5552	4645 (83.7)	556 (12.0)	505	8 (1.6)	26 (5.1)
	70+	2830	2352 (83.1)	274 (11.7)	235	8 (3.4)	15 (6.4)

CRC: Colorectal cancer; IR or HR: Intermediate or high risk.

a Age at initiation of repeat screening round.

The interval between the initial and the repeat gFOBT screen was two years for most participants (97.0%). Excluding participants with an interval between consecutive screening rounds of greater than two years did not affect positivity or PPV substantially (data not shown). Positivity remained 11.0% in participants with a previous diagnostic test outcome of low risk adenomas or normal colon and rectum, and 12.0% in participants with a previous outcome of abnormal colorectal findings. The PPV for colorectal cancer remained at 2.2% and 1.6% in participants with a previous outcome of low risk adenomas and abnormal colorectal findings, respectively, and increased marginally from 1.3% to 1.4% in participants with a previous diagnostic outcome of normal colon and rectum.

Among the 3572 individuals who tested positive again at their repeat gFOBT, and attended another diagnostic investigation, 3261 (91.3%) did not have cancer, or adenomas requiring surveillance, detected; 632 of these 3261 participants were invited for a further gFOBT screening round between August 2006 and April 2013. Uptake of this further gFOBT screen was 88.4% (95% CI: 85.7–90.8%). The positivity in this further round was even higher, at 22.9% (95% CI: 19.5–26.6%), yet upon diagnostic investigation of 115 of the 128 gFOBT positive individuals, no cancers were found, and only four participants had intermediate or high risk adenomas detected (Table 3).

**Table 3.** Outcomes of a further screening round after two preceding rounds both with an outcome of positive gFOBT and diagnostic investigation negative for cancer or intermediate/high risk adenomas (*n* = 632).

Result of diagnostic investigation in preceding round	Outcome of repeat screening round					
	Invited	Adequately screened (%)	Positive gFOBT (%)	Attended diagnostic investigation	CRC	IR or HR adenomas (PPV, %)
No cancer or IR/HR adenomas	632	559 (88.4)	128 (22.9)	115	0	4 (3.5)
Normal colon & rectum	245	209 (85.3)	46 (22.0)	40	0	1 (2.5)
Low risk adenomas	128	120 (93.8)	32 (26.7)	31	0	3 (9.7)
Other abnormal	259	230 (88.8)	50 (21.7)	44	0	0 (0)

CRC: Colorectal cancer; IR or HR: Intermediate or high risk.

## Discussion

Among individuals with a previous positive gFOBT and diagnostic investigation negative for cancer or adenomas requiring surveillance, repeat gFOBT screening positivity was high (11.3%) and the PPV for colorectal cancer (1.7%), or intermediate/high risk adenomas (7.0%), was low. Conversely, among individuals with a previous negative gFOBT, positivity at repeat screening was 1.3%, the PPV for cancer was 9.1%, and the PPV for intermediate/high risk adenomas was 25.6%.

More generally in the BCSP, positivity is lower and PPV for cancer or intermediate/high risk adenomas is higher than reported here after positive gFOBT and negative diagnostic investigation. In a study of 62,099 individuals invited for gFOBT screening in the BCSP Southern Hub, positivity was 1.2% in the first round and 1.7% in the second round of screening.^16^ The same study reported a PPV for cancer of 10.9% in the first round and 8.4% in the second round.^16^ Another study reported that among the first 2.1 million individuals ever invited to the BCSP, 17,518 attended a diagnostic investigation following a positive gFOBT, the PPV for cancer was 10.1%, and the PPV for intermediate/high risk adenomas was 27.2%.^3^

The considerably higher positivity and lower PPV after previous positive gFOBT and negative diagnostic investigation indicates that some individuals are prone to repeat positive gFOBTs in the absence of colorectal cancer or intermediate/high risk adenomas. Further evidence for this comes from the 632 individuals invited to a further gFOBT screening round after two consecutive rounds of positive gFOBT and diagnostic investigation negative for cancer or intermediate/high risk adenomas. Among this group, positivity was even higher, at 22.9%; no cancers were detected, and only four individuals were found to have intermediate or high risk adenomas. The exact cause of repeat positive gFOBTs in the absence of cancer and intermediate or high risk adenomas is unclear. The consistently high positivity and low PPVs across age groups, sex, and outcome of initial investigation (normal, low risk adenomas, or other abnormal) indicates a cause to some extent independent of these factors.

Guaiac faecal occult blood tests detect the peroxidase activity of haem, a component of haemoglobin found in blood in stool. One potential explanation for repeat false-positive gFOBTs is that some participants have an alternative source of chronic gastrointestinal bleeding, such as upper gastrointestinal lesions.^17^ It is also thought that non-steroidal anti-inflammatory drugs, such as aspirin, may cause increased gastrointestinal bleeding and thereby increase the number of false-positive gFOBTs.^18–20^ A highly specific non-invasive test for colorectal cancer, which is not based on the detection of occult bleeding, could be very useful for further screening of patients following positive gFOBT and negative colonoscopy. Unfortunately, no such test currently exists.

The consumption of red meat and high-peroxidase fruit and vegetables shortly before testing has also been linked to false-positive gFOBTs.^21^ In the BCSP, participants are not requested to make any dietary restrictions, given the potential negative effect of this on uptake and uncertain efficacy of dietary restriction in preventing false positivity.^3^

A number of other studies using smaller datasets have similarly found positivity to be high and PPV for cancer to be low for a repeat gFOBT or FIT screen in participants with a previous negative colonoscopy.^7–15^ Carrera and colleagues examined the outcomes of repeat gFOBT screening within the Scottish screening pilot among participants with no neoplasia on diagnostic investigation after positive gFOBT.^9^ In a second round of screening in this group, positivity was 17.4% (157/904 participants) and six participants had cancer (PPV 3.8%). In the third round of screening, 84 individuals who had a positive gFOBT and negative colonoscopy in both the first and second round were invited. Positivity was 25.6% in this group and no cancers or adenomas were detected.

Although the PPV of a repeat screen following positive gFOBT and negative diagnostic investigation was low, the high positivity rate meant that the yield of colorectal cancer and the yield of intermediate/high risk adenomas, as a proportion of participants adequately screened, were higher than after negative gFOBT. However, due to the low PPV, many participants undergoing repeat screening will be subject to additional unnecessary diagnostic investigation, with the accompanying risk of adverse events, such as colonoscopic perforation, and the potential for psychological distress.^22–25^ Furthermore, additional colonoscopies will increase cost and place additional demand on overburdened endoscopy services.

Missed lesions and incomplete resection are major causes of colorectal cancer detected soon after colonoscopy.^26^ Ensuring that colonoscopies are of high-quality, with good bowel preparation, cecal intubation, high adenoma detection rates, and complete resection of advanced lesions, is crucial to minimizing occurrences of post-colonoscopy colorectal cancer.

## Conclusions

In participants undergoing repeat screening following a previous positive gFOBT and negative colonoscopy, test positivity is high and PPV for colorectal cancer is low. Though colorectal cancers are diagnosed in these participants, it comes at a cost of an increase in the number of colonoscopies needed.
